# Supplementary material for: MetaRibo-Seq measures translation in microbiomes
Source: Nat Commun. 2020 Jun 29;11:3268. doi: 10.1038/s41467-020-17081-z (PMC7324362; doi:10.1038/s41467-020-17081-z)
Supplement: Supplementary file 10 — Supplementary Data 7 [file 41467_2020_17081_MOESM10_ESM.zip › File2/Confidence_High_Taxonomy/339726_out.krona.html]

Javascript must be enabled to view this page.

members
magnitude
magnitudeUnassigned
count
unassigned
taxon
rank

339726\_out

2

2759
1
superkingdom

4751
1
kingdom

1
451864
subkingdom

phylum
4890
1

subphylum
1
147538

147545
1
class

451871
1
subclass

1
5042
order

1131492
1
family

5052
1
genus


SRS1041145\_contig\_number\_contig-100\_937.185010
species
1
40382


SRS051031\_contig\_number\_contig-100\_5209.92468
1
